# Bird’s Elements of Natural Philosophy

**Published:** 1848

**Authors:** 


### ARTICLE IV.

*Elements of Natural Philosophy: being an Experimental Introduction to the Study of the Natural Sciences.* By Golding Bird, A.M., M.D., F.R.S., F.L.S., etc., etc. With 372 illustrations. From the Revised and Enlarged third London Edition. pp. 402, 12mo. Philadelphia: Lea & Blanchard. 1848. (From the Publishers.)

The celebrity of the author is alone sufficient to recommend the above work to those who have noticed his contributions to medical science in the medical periodicals for the last few years—still more so to those who have perused his other works on subjects connecting physical science with medicine. The volume before us is intended principally as a text book for medical students attending the lectures of physics always preceding the chemical course in our medical schools. We heartily recommend it to such as the best work for its object which has yet appeared; indeed, excepting that future discoveries would require to be added, we can scarcely anticipate the appearance of another equally clear, concise, and well illustrated.

J. V. Z. B.

